# Magnetic Resonance Imaging of Cyclops Lesion in Patients With Non-reconstructed Anterior Cruciate Ligament

**DOI:** 10.7759/cureus.62894

**Published:** 2024-06-22

**Authors:** Tushar Kalekar, Sai pavan Kumar, Tejvir Singh, Apurvaa Pachva, Nikhith Soman

**Affiliations:** 1 Department of Radiology, Dr. D. Y. Patil Medical College, Hospital and Research Centre, Pune, IND

**Keywords:** anterior intercondylar region, knee extension, arthrofibrosis, anterior cruciate ligament, cyclops

## Abstract

Introduction

The term cyclops lesion refers to localized anterior fibrosis, which is the abnormal proliferation of fibrous tissue in a joint that develops in the anterior aspect of the intercondylar notch. It is a known cause of extension loss of the knee after anterior cruciate ligament (ACL) reconstruction; however, it can be found in patients who have not undergone any surgical repair of the ACL. The term “cyclops lesion” was given based on the arthroscopic appearance of the fibrous nodule and vessels that resemble an eye. The purpose of this study is to highlight the existence of cyclops lesions in non-operated knees.

Methods

We conducted a study on 10 patients who were subjected to an MRI knee in a Siemens Magnetom Vida 3 Tesla (Erlangen, Germany) machine. We retrospectively analyzed all 10 cases in our institution from July 2021 to March 2022. These subjects had a previous history of trauma, and they presented with complaints of pain and difficulty in knee extension but no history of previous ligament repair. All patients underwent an MRI examination. When a cyclops lesion was revealed on MR imaging, the signal-intensity characteristics, location, and size were documented.

Results

There were a total of 10 patients included in the study, of whom eight were males and two were females. The most common clinical presentation in all our cases was difficulty in the extension of the knee, while there was associated instability and difficulty in walking in some patients. There was a demonstrable cyclops lesion near the tibial attachment of ACL in eight (80%) patients, whereas it was found to be located just lateral to the anterior intercondylar notch in the rest of the two (20%) patients.

Conclusion

MRI is an effective tool to evaluate unexplained pain, functional limitations, and limited range of motion in patients with suspected arthrofibrosis. MRI also helps determine the extent of fibrosis involvement and excludes other complications that may have a similar clinical picture.

## Introduction

Cyclops lesion is a painful anterior knee mass, also known as localized anterior arthrofibrosis. It comprises heterogeneous localized metaplasia composed of fibrous granulation tissue [1]. Cyclops lesion is a common complication in post-anterior cruciate ligament (ACL) reconstruction. The Cyclops lesion is a fibrovascular nodule that may be pedunculated or not. A cyclops-like look is created by the arthroscopic appearance and the overlaying blood vessels. The tibial anterolateral aspect is where the ACL graft is most frequently placed, and the cyclops lesion is commonly situated anterior to the graft. The nodule has a size range of 3 to 12 millimeters. It has been recently identified in people who have suffered ACL damage but have not received reconstructive surgery [2].

Patients with acute anterior cruciate ligament (ACL) injuries, which are mostly brought on by pain, swelling, and muscle spasms, frequently have diminished knee extension. Recovery normally takes a few weeks, with a focus on restoring the full range of motion [3]. Arthroscopic treatment with removal of the nodule is the treatment of choice to regain extension movements, as even aggressive physical therapy does not have a great impact in cases of cyclops nodules [4,5].

The typical clinical symptoms of cyclops lesions include a rubbery endpoint to full extension with or without a detectable snap, an initial full range of motion that is later lost, and a rebound after manipulation into full extension. However, these outcomes could be unpredictable, making them not particularly reliable [6].

## Materials and methods

A study of a total of 10 patients was done after the informed consent of the patients, and approval of the study was given by the Institutional Review Board.

We retrospectively analyzed all 10 cases of Cyclops lesion in our institution from July 2021 to March 2022. Along with relevant clinical information received from the patient and orthopedics, magnetic resonance (MR) images were collected from the Picture Archiving and Communication System (PACS). Patients were selected based on their clinical profile and imaging appearances.

A Siemens Magnetom VIDA 3T MRI (Erlangen, Germany) machine was used to carry out the examination. The patient was placed in a lying-down position on the MRI table, and a knee surface coil was placed. The knee was kept in the surface coil with extension and external rotation of approximately 10-15 degrees. The knee was secured in the coil by centering the joint. A dedicated 18-channel transmit-receive knee coil was used.

An MRI scan was done using the following standard imaging protocol: The various imaging planes acquired in our study with relevant slice thickness and imaging settings have been described in Table 1.

**Table 1:** Sequence in knee joint MRI TR: Time to repeat (milliseconds), TE: Time to echo (milliseconds)

Image plane	TR	TE	Slice thickness	FOV
PDFS Coronal	3500	38	3 mm	130
PDFS Sagittal	3500	38	3 mm	130
PDFS Axial	3500	38	3 mm	130
T1 Coronal	530	20	3 mm	130
T2 Sagittal	2800	72	2 mm	130
T2 med2cor (Gradient)	600	14	2 mm	140

## Results

The inclusion criteria for the study involved patients with a history of knee trauma, particularly those who had anterior cruciate ligament (ACL) injury with no history of ligament repair. The inclusion criteria have specified a certain age range and duration since trauma and clinical symptoms related to knee extension difficulties and pain, with or without concomitant meniscal lesions, have been part of the inclusion criteria.

On the other hand, the exclusion criteria may have involved patients with pre-existing knee conditions unrelated to trauma, those with incomplete medical records or imaging studies, and patients who had undergone previous knee surgeries. 

The study group consists of eight males and two females, with six patients in the age group of 18 to 30 years, three patients in the age group of 31 to 45 years, and one patient above 45 years old. All patients had a history of trauma, with some experiencing twisting injuries and others having injuries in road traffic accidents. All patients sustained trauma at least three months before their MRI examination, with the maximum time from trauma being two years. The most common clinical presentation among all cases was pain while walking, with associated instability and difficulty in walking in some patients. The study found that the commonly affected knee movement was difficulty in knee extension. Examination findings include loss of full extension (15-20° extension lag) and tender medial joint line. All patients had a complete tear of the ACL.

Anterior translation of the tibia relative to the femur of 3 mm is noted in five patients, 4 mm in three patients, and 5 mm in two patients.

There was an injury to the medial meniscus in eight patients (80%). A grade I signal in the posterior horn was seen in three patients (30%), a grade II signal in the posterior horn in four patients (40%), and a complex tear involving the posterior horn in one patient (10%), respectively. A normal medial meniscus was seen in two patients (20%).

Sex and age of all the patients, duration since trauma, movements affected, and status of the anterior cruciate ligament (ACL), posterior cruciate ligament (PCL), medial meniscus (MM) of the affected knee, and anterior translation of the tibia (in mm), which were included in the study, are listed in Table 2.

**Table 2:** Sex and age of all the patients, duration since trauma, movements affected, anterior translation of tibia, status of the ACL, PCL, and MM of the affected knee. ACL: anterior cruciate ligament, PCL: posterior cruciate ligament

Sex	Age	Duration since trauma	Movements affected	Status of ACL	Anterior translation of tibia (mm)	Status of PCL	Status of Medial meniscus
Male	27	2 months	None	Complete tear	3	Buckling	Grade I signal in posterior horn
Male	19	3 months	Knee extension	Complete tear	3	Buckling	Normal
Male	25	3 months	Knee extension	Complete tear	3	Normal	Grade I signal in posterior horn
Male	36	24 months	Knee extension	Complete tear	4	Normal	Grade II signal in body
Female	30	7 months	None	Complete tear	5	Normal	Grade II signal in posterior horn
Male	47	8 months	Knee extension	Complete tear	5	Normal	Grade II signal in posterior horn
Male	30	6 months	None	Complete tear	3	Normal	Grade I signal in posterior horn
Male	40	4 months	Knee extension	Complete tear	4	Normal	Complex tear in posterior horn
Male	18	2 months	Knee extension	Complete tear	4	Buckling	Grade II signal in body
Female	32	6 months	Knee extension	Complete tear	3	Buckling	Normal

On MRI, a soft-tissue nodule is seen anteriorly or anterolaterally in the intercondylar notch near the tibial insertion of the anterior cruciate ligament. The lesions showed an intermediate signal on PDFS images in all patients. On T2 weighted imaging, eight (80%) patients showed an intermediate hypointense signal with respect to the skeletal muscle, whereas an isointense signal was noted in two (20%) patients. Cyclops lesions were examined for size in two dimensions: axial (width), and sagittal (depth). We noted the locations of lesions relative to the attachment of ACL on proton density and T2-weighted images. There was a demonstrable PDFS intermediate signal lesion in the region near the tibial attachment of the ACL in the anterior intercondylar notch region, in eight patients (80%), whereas it was found to be located lateral to the anterior intercondylar notch in the rest of the two patients (20%). Treatments range from conservative management to physiotherapy and arthroscopy.

Table 3 depicts the size and location of the lesion along with signal characteristics on proton density fat-saturated images (PDFS), T2WI, and corresponding treatment for each case. 

**Table 3:** Depicts the size and location of the lesion along with signal characteristics on PDFS and T2WI. PDFS: proton density fat-saturated images (PDFS)

Size of lesion	Location of lesion	Signal on PDFS	Signal on T2WI	Treatment
12 x 8 mm	Anterior intercondylar region	Intermediate	Hypointense	Conservative
12 x 9 mm	Anterior intercondylar region	Intermediate	Hypointense	Conservative
15 x 12 mm	Anterior intercondylar region	Intermediate	Hypointense	Physiotherapy
7 x 6 mm	Anterior intercondylar region	Intermediate	Isointense	Conservative
12 x 11 mm	Lateral to anterior notch	Intermediate	Isointense	Conservative
10 x 7 mm	Anterior intercondylar region	Intermediate	Hypointense	Arthroscopy
6 x 5 mm	Anterior intercondylar region	Intermediate	Hypointense	Physiotherapy
14 x 9 mm	Anterior intercondylar region	Intermediate	Hypointense	Conservative
12 x 9 mm	Anterior intercondylar region	Intermediate	Hypointense	Conservative
9 x 6 mm	Lateral to anterior notch	Intermediate	Hypointense	Conservative

**Figure 1:**
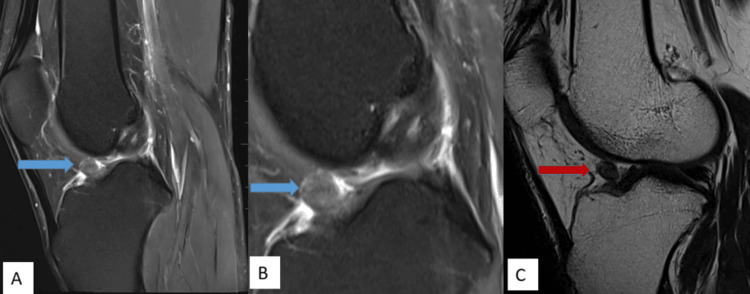
A fairly well-defined lesion measuring approx. 10 x 7 mm is noted in the anterior intercondylar notch in relation to the lower third of ACL, showing intermediate signal intensity on PDFS sagittal (A) and magnified PDFS sagittal (B) images (blue arrow) with a hypointense signal on T2W sagittal images (C) (red arrow). PDFS: proton-density fat-suppressed

**Figure 2:**
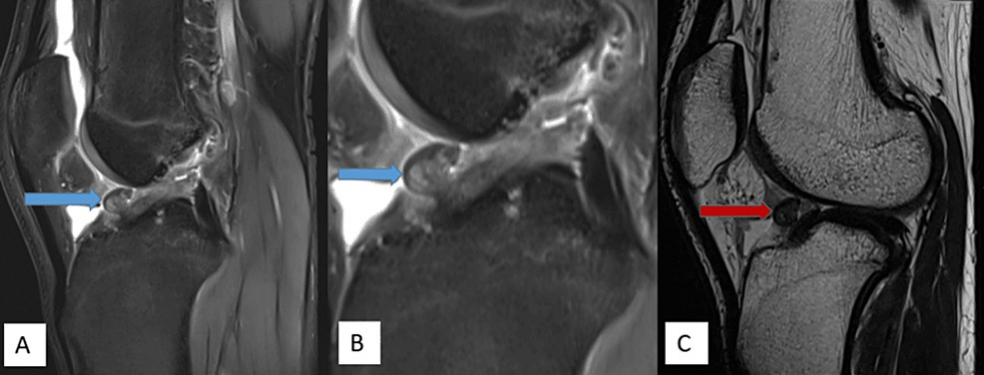
A well-defined nodular lesion measuring approx. 12 x 9 mm is noted in the deep Hoffa’s fat pad, anterior intercondylar notch near ACL attachment showing intermediate signal intensity on PDFS sagittal (A) and magnified PDFS sagittal (B) images (blue arrow) with hypointense signal on T2W sagittal images (C) (red arrow). ACL: anterior cruciate ligament, PDFS: proton density fat-saturated

**Figure 3:**
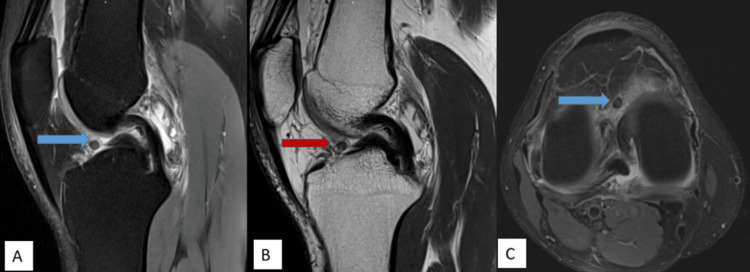
A well-defined, rounded PDFS hypointense structure measuring approx. 6 x 5 mm is noted in the anterior intercondylar region on PDFS sagittal (A) and PDFS axial (C) images (blue arrow). It is showing a hypointense T2W signal on T2WI sagittal (B) images (red arrow). PDFS: proton density fat-saturated

**Figure 4:**
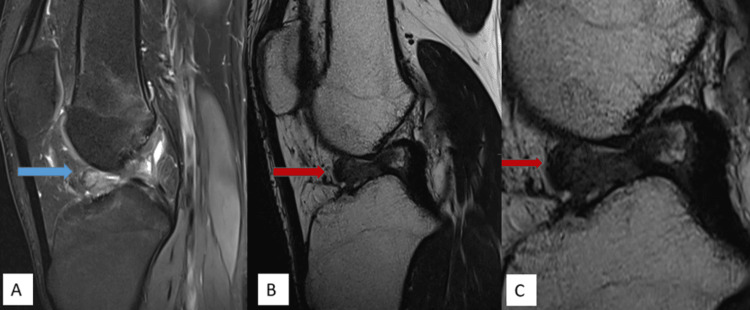
A well-defined soft tissue intensity lesion measuring approx. 12 x 9 mm, showing intermediate PDFS hyperintense signal on PDFS sagittal (A) (blue arrow) and T2 hypointense signal on T2WI sagittal (B) and magnified T2WI sagittal images (red arrow), is noted in the anterior intercondylar notch anterior to the tibial attachment of the anterior cruciate ligament. PDFS: proton density fat-saturated

**Figure 5:**
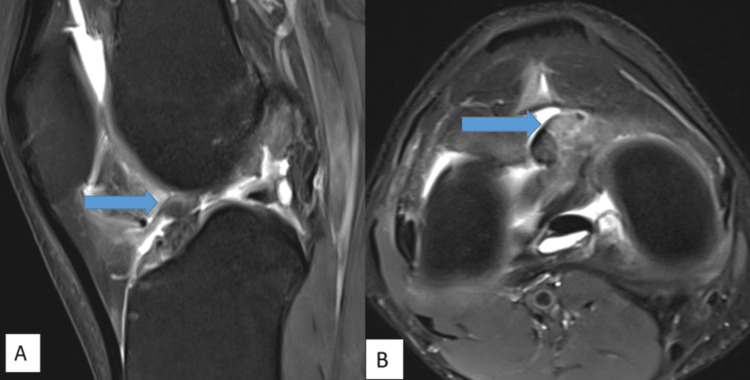
A fairly well-defined PDFS hypointense structure measuring approximately 12 x 9 mm is noted in deep Hoffa’s fat pad anterior to expected ACL attachment on PDFS sagittal (A) and PDFS axial (B) images ACL: anterior cruciate ligament, PDFS: proton density fat-saturated

MRI imaging features are shown in Figures 1-5.

## Discussion

Cyclops lesions are not commonly described in the literature as developing in non-operated knees that originate from the native ACL as a consequence of its tear. The Cyclops lesion is a nodule of fibrovascular tissue. The arthroscopic appearance, with its overlying blood vessels, gives the appearance of a “cyclops.” We conducted a study on 10 patients with an age range of 18-45 years. All patients had ACL injuries with no history of ligament repair. There was a demonstrable PDFS intermediate signal lesion in the region near the tibial attachment of the ACL in the anterior intercondylar notch region and lateral to the anterior intercondylar notch. According to our study, Cyclops lesions can also arise as a scar reaction to trauma or as a reactive fibroproliferative process that occurs after the native ACL fibers rupture.

Cyclops lesions were most commonly iatrogenic and were first noted to develop following ACL reconstruction, with a typical period of 16 weeks between ACL restoration and arthroscopy. The most commonly encountered typical clinical features are pain and loss of extension movement of the knee joint [7,8]. Symptoms, pathology, and imaging features of cyclops lesions develop after long periods, even up to 24 years after trauma, as described in recent papers [9,10].

Patients with trauma to the knee in childhood reported difficulty in knee extension and restriction of movements several years following the trauma [11]. Relevant history and clinical examination in such cases can offer great help in looking for causes of painful knee and restricted knee movements and then further recommending patients for detailed imaging.

Cyclops syndrome, which is difficulty in knee extension due to an underlying cyclops nodule, was originally identified by Jackson and Schaefer in 1990 in patients who had previously undergone patellar autograft anterior cruciate ligament (ACL) reconstruction [12]. Two to fifteen percent of all ACL (anterior cruciate ligament) replacement patients are unable to regain full knee extension [13,14]. In a few of these patients, a cyclops lesion has generated a mechanical block that precludes full extension [15,16]. When there is associated difficulty in knee extension with an audible, palpable clunk during terminal extension, it is known as cyclops syndrome. Cyclops syndrome has been mentioned as a side effect of ACL restoration in earlier published publications. McMahon et al. [17] first reported a study on four cases of Cyclops syndrome following an ACL rupture that wasn't fixed surgically. The occurrence of Cyclops syndrome following ACL rupture without surgical reconstruction was also documented by Tonin et al. [18]. In all of these cases, the knee extension was reduced within two months of the injury.

They proposed the theory that the arthroscopy-discovered nodule developed as a result of a normal fibroproliferative process that developed from the drilled-out debris or occurred as a result of frayed graft fibers in patients who have had an ACL reconstruction. Therefore, it was believed that the granulation and fibroproliferative process caused the Cyclops lesion.

In post-ACL reconstruction patients, the microtrauma pathophysiology described by Marzo et al. presumably plays a significant role in the development of cyclops lesions [19].

When cyclops lesions appear after trauma, even remote trauma, the pathologic and imaging results are comparable, supporting a similar mechanism. We propose that the development of cyclops nodules in trauma patients with clinically or radiologically intact ACLs is likely also caused by microtrauma to subclinically torn ACL fibers [2]. After graft impingement, cyclops lesions are the second most frequent reason for limited knee extension [20].

Cyclops nodules do not always lead to Cyclops syndrome. Muellner et al. [21], who conducted a study on patients whose ACLs had been repaired using patellar tendon autografts, distinguished between two forms of cyclops nodules: real cyclops nodules and cyclopoid scars. These nodules occur as a result of two distinct histomorphologic processes.

True cyclops nodules have bone or cartilaginous debris inside and are hard in consistency. They are frequently linked to clinical Cyclops syndrome. Cyclopoid scars, on the other hand, are made entirely of fibroproliferative tissue. Cyclops syndrome is uncommon in cyclopoid scars because the nodule is soft and compressed by the bone, preventing full extension [2].

The cyclops lesions in previously done studies showed similar MR imaging signal characteristics, with a predominance of heterogeneous or low-signal intensity nodules on proton density fat-suppressed (PDFS) and T2 fat-saturated images. Focal pigmented villonodular synovitis presenting as a focal heterogeneous low-signal-intensity nodule adjacent to the knee joint is considered a close differential diagnosis. Furthermore, there is a histological overlap between cyclops nodules and nodular synovitis, both of which show collagen fiber stroma with varying hemosiderin deposition. MR imaging in such dilemmas acts as the most useful imaging tool in differentiating between cyclops nodules and nodular synovitis, with proximity to the ACL or ACL remnant going in favor of cyclops nodules. Clinical history and examination can also be helpful in patients where there is a dilemma in the imaging findings [2].

In all patients with a previous history of trauma, the integrity of the ACL should be carefully looked for, as should direct signs of disruption, such as an abnormal hyperintense signal within the ACL, which can be either focal or generalized. Along with this, some other signs, such as a wavy, irregular contour of the ligament and non-visualization of the ACL, were used to determine the extent of an ACL tear [22,23].

However, there is currently no evidence to support differences in MRI appearance (size, signal intensity, location, etc.) between symptomatic and asymptomatic lesions [24]. Other differentials of anterior knee masses causing pain and difficulty in extension include infrapatellar fat neoplasms, para-articular chordomas, infrapatellar ganglion cysts, etc. [1].

MR imaging, however, is the modality of choice in the detection of cyclops lesions, provides a noninvasive means of detection, and aids in the identification of individuals who require further management as MRI can distinguish bone and soft tissue detail [6]. MR imaging provides an upper hand in diagnostic evaluation as it talks about other associated injuries with regard to the status of the posterior cruciate ligament, medial and lateral menisci, along collateral ligaments.

Limitations

The study focuses on a specific type of knee injury (cyclops lesions) and may not cover all possible knee conditions or injuries that could present with similar symptoms. Our study included a small number of patients, which prevented a statistical analysis from being performed. Most studies that we identified were case reports or small case series. Hence, a systematic review could not be performed. The study may not address the effectiveness of different treatment options for cyclops lesions or provide comprehensive management strategies. This study was retrospective, and no arthroscopic or histological correlation has been assessed, as the diagnosis of Cyclops was solely a radiological one.

## Conclusions

Cyclops lesion, which is usually a common complication in post-ACL reconstruction, can be seen in patients with no previous ACL reconstruction but a previous ACL injury history. Most of the patients had difficulty walking, and knee extension was the most commonly affected movement. MRI is effective as a tool to evaluate unexplained pain, limited range of motion, and functional limitation in patients in whom arthrofibrosis is suspected. MRI also helps in establishing the extent of involvement by fibrosis and to exclude other complications that may have a similar clinical presentation. Thus, timely diagnosis of cyclops in such patients can benefit the patient and help in further management.

## References

[1] Flores DV, Mejía Gómez C, Pathria MN (2018). Layered approach to the anterior knee: normal anatomy and disorders associated with anterior knee pain. Radiographics.

[2] Runyan BR, Bancroft LW, Peterson JJ, Kransdorf MJ, Berquist TH, Ortiguera CJ (2007). Cyclops lesions that occur in the absence of prior anterior ligament reconstruction. Radiographics.

[3] Nakagawa T, Hiraoka H, Fukuda A (20061). Symptomatic cyclops lesion after rupture of the anteromedial bundle of the anterior cruciate ligament. Journal of Orthopaedic Science.

[4] Shelbourne KD, Johnson GE (1994). Outpatient surgical management of arthrofibrosis after anterior cruciate ligament surgery. Am J Sports Med.

[5] Mariani PP, Ferretti A, Conteduca F (19921). Arthroscopic treatment of flexion deformity after ACL reconstruction. Jr Arthr Rel Surg.

[6] Bradley DM, Bergman AG, Dillingham MF (2000). MR imaging of cyclops lesions. AJR Am J Roentgenol.

[7] Delcogliano A, Franzese S, Branca A, Magi M, Fabbriciani C (1996). Light and scan electron microscopic analysis of cyclops syndrome: etiopathogenic hypothesis and technical solutions. Knee Surg Sports Traumatol Arthrosc.

[8] Dandy DJ, Edwards DJ (1994). Problems in regaining full extension of the knee after anterior cruciate ligament reconstruction: does arthrofibrosis exist?. Knee Surg Sports Traumatol Arthrosc.

[9] Petsche TS, Hutchinson MR (19991). Loss of extension after reconstruction of the anterior cruciate ligament. Jr Amer Aca Ortho Surg.

[10] Veselko M, Rotter A, Tonin M (20001). Cyclops syndrome occurring after partial rupture of the anterior cruciate ligament not treated by surgical reconstruction. Jr Arth Rel Sur.

[11] Irisawa H, Takahashi M, Hosokawa T, Nagano A (2007). Cyclops syndrome occurring after chronic partial rupture of the anterior cruciate ligament without surgical reconstruction. Knee Surg Sports Traumatol Arthrosc.

[12] Jackson DW, Schaefer RK (19901). Cyclops syndrome: loss of extension following intra-articular anterior cruciate ligament reconstruction. Jr Arthr Rel Sur.

[13] Buss DD, Warren RF, Wickiewicz TL (1993). Arthroscopically assisted reconstruction of the anterior cruciate ligament with use of autogenous patellar-ligament grafts. Results after twenty-four to forty-two months. JBJS.

[14] Graf BK, Ott JW, Lange RH, Keene JS (1994). Risk factors for restricted motion after anterior cruciate reconstruction. Orthopedics.

[15] Watanabe BM, Howell SM (1995). Arthroscopic findings associated with roof impingement of an anterior cruciate ligament graft. Amer Jr Spo Med.

[16] Cosgarea AJ, DeHaven KE, Lovelock JE (1994). The surgical treatment of arthrofibrosis of the knee. Am J Sports Med.

[17] McMahon PJ, Dettling JR, Yocum LA (19991). The cyclops lesion: a cause of diminished knee extension after rupture of the anterior cruciate ligament. Jr Arthr Rel Sur.

[18] Tonin M, Saciri V, Veselko M, Rotter A (2001). Progressive loss of knee extension after injury. Cyclops syndrome due to a lesion of the anterior cruciate ligament. Am J Sports Med.

[19] Marzo JM, Bowen MK, Warren RF (19921). Intraarticular fibrous nodule as a cause of loss of extension following anterior cruciate ligament reconstruction. Jr Arthr Rel Sur.

[20] Recht MP, Piraino DW, Cohen MA, Parker RD, Bergfeld JA (1995). Localized anterior arthrofibrosis (cyclops lesion) after reconstruction of the anterior cruciate ligament: MR imaging findings. AJR Am J Roentgenol.

[21] Muellner T, Kdolsky R, Grossschmidt K, Schabus R, Kwasny O, Plenk H Jr (1999). Cyclops and cyclopoid formation after anterior cruciate ligament reconstruction: clinical and histomorphological differences. Knee Surg Sports Traumatol Arthrosc.

[22] Reicher MA, Hartzman S, Bassett LW, Mandelbaum B, Duckwiler G, Gold RH (1987). MR imaging of the knee. Part I. Traumatic disorders. Radiology.

[23] Lee JK, Yao L, Phelps CT, Wirth CR, Czajka J, Lozman J (1988). Anterior cruciate ligament tears: MR imaging compared with arthroscopy and clinical tests. Radiology.

[24] Kambhampati SB, Gollamudi S, Shanmugasundaram S, Josyula VV (2020). Cyclops lesions of the knee: a narrative review of the literature. Orthop J Sports Med.

